# Atypical cerebral fat embolism syndrome in an elderly patient with comorbidities following fracture: A case report

**DOI:** 10.1097/MD.0000000000047052

**Published:** 2026-01-16

**Authors:** Yu Wei, Hanyu Zhong, Guanghong Xiang

**Affiliations:** a The School of Clinical Medicine, Hunan University of Chinese Medicine, Yuelu District, Changsha, Hunan Province, China; b Department of Neurology, Hunan Second Provincial People's Hospital (Brain Hospital of Hunan Province), Changsha, Hunan Province, China.

**Keywords:** case report, cerebral fat embolism, fat embolism syndrome, multiple fractures

## Abstract

**Rationale:**

Cerebral fat embolism (CFE) is a rare but serious complication of fractures, most commonly affecting young males. However, atypical cases in elderly patients with underlying comorbidities are easily misdiagnosed.

**Patient concerns:**

We report a case of a 61-year-old male patient with diabetes and hypertension who developed impaired consciousness and fever on the 2nd day after sustaining multiple fractures in a traffic accident. His condition deteriorated after receiving local treatment.

**Diagnoses:**

CFE was diagnosed based on the characteristic “starry sky sign” on brain magnetic resonance imaging–diffusion-weighted imaging after excluding other common causes of stroke.

**Interventions:**

The patient received comprehensive supportive care, including anticoagulation, hyperbaric oxygen therapy, and early surgical fracture fixation.

**Outcomes:**

The patient’s level of consciousness improved. Following stabilization, rehabilitation therapy was initiated.

**Lessons:**

For atypical CFE patients lacking respiratory symptoms and skin hemorrhagic spots, early diagnosis is challenging and prone to being missed. Only through early detection and prompt, aggressive treatment can prognosis be improved.

## 1. Introduction

**Figure 1.**
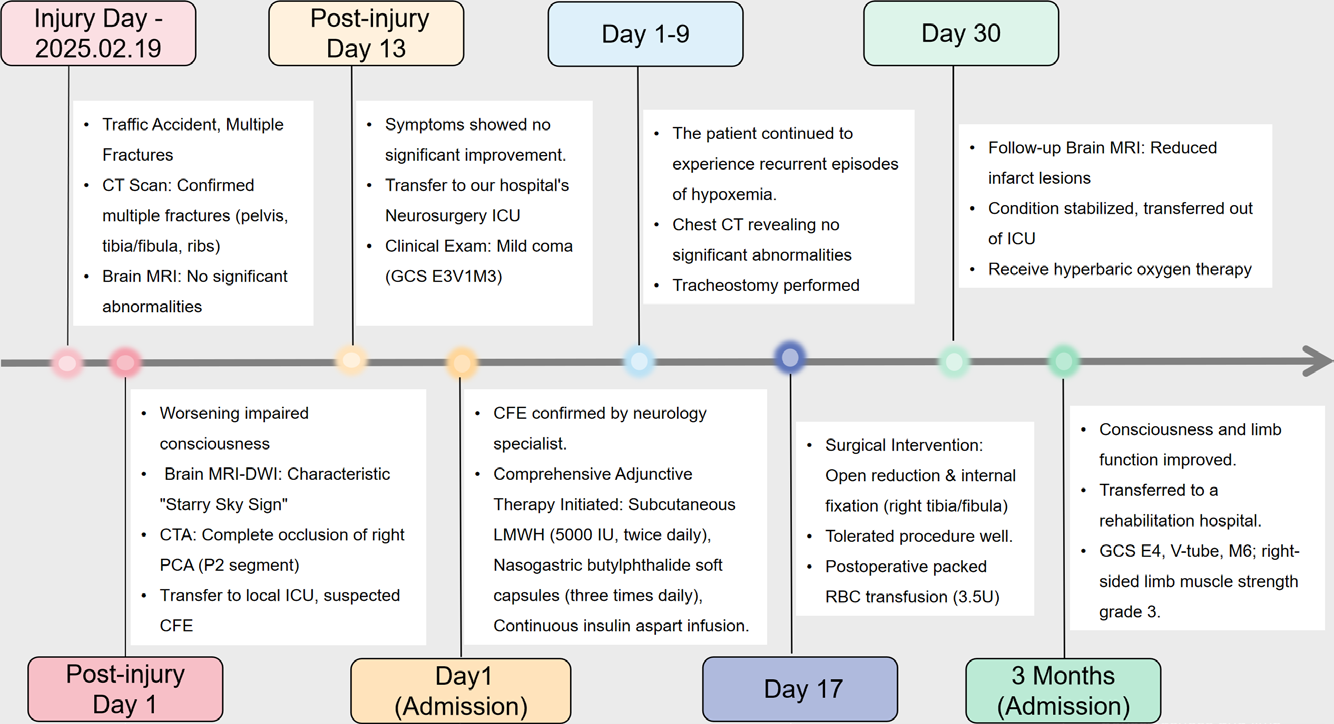
Timeline of the clinical course, diagnostic findings, and interventions for a patient with CFE following multiple fractures. Key events include the initial trauma, symptom onset, critical neuroimaging findings, and therapeutic measures. CFE = cerebral fat embolism, DWI = diffusion-weighted imaging, GCS = Glasgow Coma Scale, HBOT = hyperbaric oxygen therapy, LMWH = low-molecular weight heparin, PCA = posterior cerebral artery.

Fat embolism syndrome (FES) is a clinical syndrome in which fat particles, typically originating from bone marrow or soft tissues, enter the bloodstream, occlude small vessels, and trigger a systemic inflammatory response.^[1]^ It is primarily characterized by the classic triad of respiratory failure, neurological dysfunction, and cutaneous ecchymosis, and carries a high mortality risk due to potential multi-organ failure.^[2]^ Cerebral fat embolism (CFE) is a rare subtype of FES that predominantly affects young males following fractures and poses significant diagnostic challenges. Although CFE cases are relatively common in the literature, reports involving elderly patients with multiple comorbidities are scarce. This report describes the diagnosis and management of an elderly patient with multiple comorbidities who developed atypical CFE following multiple fractures. The patient’s clinical course is illustrated in the timeline (Fig. 1).

## 2. Case information

### 2.1. Patient information

A 61-year-old Han Chinese male, retired, with a 40-year history of smoking. He was admitted to our hospital with “13 days of generalized pain and 12 days of impaired consciousness caused by a car accident.” He had a 6-year history of type 2 diabetes and hypertension, with long-term medication use (including nifedipine sustained-release tablets, amlodipine besylate tablets, metformin hydrochloride tablets, and glimepiride sustained-release tablets), maintaining stable blood glucose and blood pressure control. He had no history of stroke, atrial fibrillation, or bleeding tendency.

### 2.2. Diagnostic assessment

**Figure 2.**
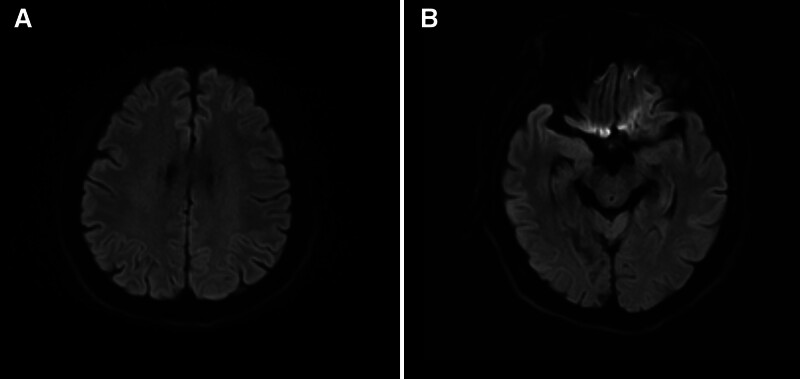
Baseline brain MRI obtained on the day of trauma. DWI sequences at the level of the cerebral ventricular body (A) and the suprasellar cistern (B) show no evidence of abnormal diffusion-restricted foci. DWI = diffusion-weighted imaging, MRI = magnetic resonance imaging.

**Figure 3.**
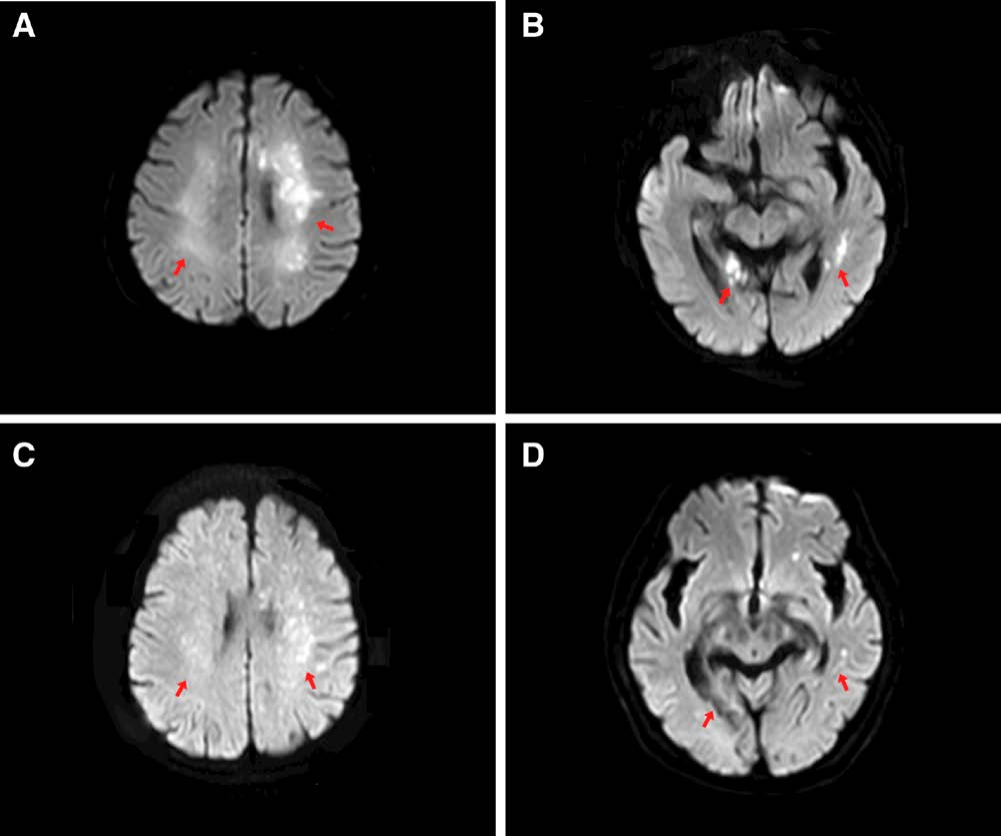
Evolution of CFE on brain MRI. (A, B) Brain MRI performed on the 1st day of neurological deterioration (day 2 post-injury). DWI sequences reveal numerous punctate hyperintense foci, some coalescing into patchy areas, distributed throughout the bilateral frontal, temporal, parietal, and occipital lobes, demonstrating the characteristic “starry sky” sign. The red arrows point to a selection of these characteristic diffuse punctate lesions. (C, D) Follow-up MRI (day 43 post-injury). DWI sequences show a significant reduction in both the number and extent of the hyperintense foci in the bilateral frontal, temporal, parietal, and occipital lobes, consistent with the patient’s clinical improvement. The red arrows point to these changed areas. CFE = cerebral fat embolism, DWI = diffusion-weighted imaging, MRI = magnetic resonance imaging.

**Figure 4.**
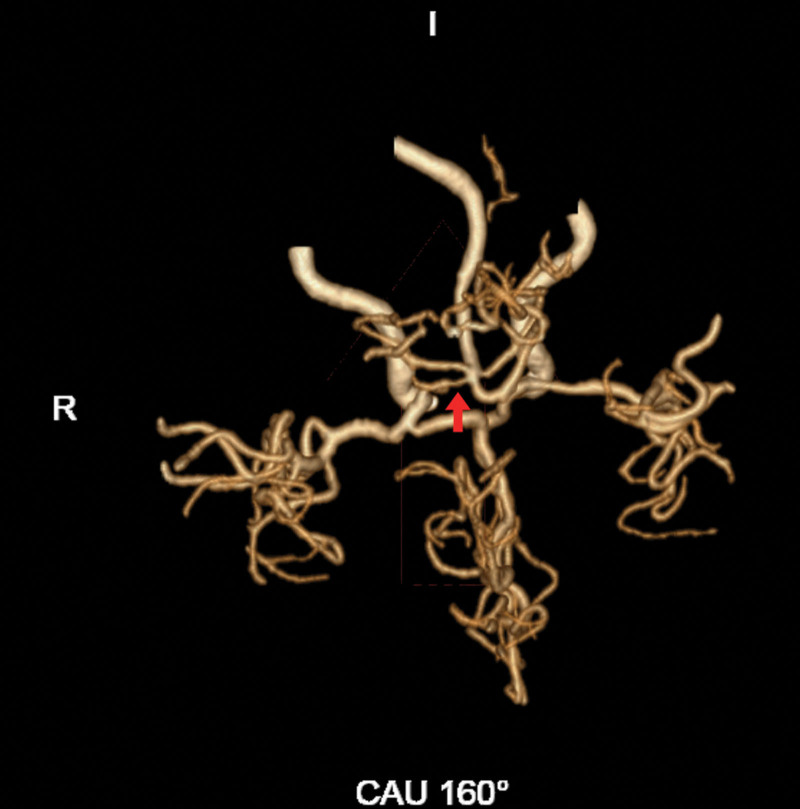
Cerebral CTA performed on the 1st day of neurological deterioration (day 2 post-injury). Cerebral CTA reveals severe stenosis of the right PCA at the P1 segment and complete occlusion at the P2 segment. An arrow indicates the site of the P1 segment stenosis. This vascular finding was deemed an incidental, chronic preexisting condition, as the distribution of the acute cerebral infarcts was inconsistent with the vascular territory supplied by the right PCA. CTA = computed tomography angiography, PCA = posterior cerebral artery.

On February 19, 2025, the patient was involved in a collision between an electric bicycle and a motor vehicle. He was immediately transported to a local hospital, where computed tomography (CT) scans revealed multiple fractures, including the right ilium, right acetabulum, bilateral superior and inferior pubic rami, right greater trochanter, sacrum, right patella, right tibial plateau, proximal and distal tibia and fibula, and left fourth rib. Brain magnetic resonance imaging (MRI) and diffusion-weighted imaging (DWI) showed no significant abnormalities (Fig. 2). A preliminary diagnosis of multiple fractures was made, and comprehensive treatment was initiated, including daily subcutaneous injections of 4100 IU low-molecular weight heparin and immobilization of the affected limbs. On the 1st day after the injury, the patient gradually developed fever, drowsiness, right upper limb muscle weakness (grade 2), and dysphagia. Due to the patient’s deteriorating condition, brain MRI and cerebral computed tomography angiography (CTA) were immediately performed. MRI revealed multiple punctate and patchy hyperintense lesions on DWI in the bilateral semioval centers, corona radiata, periventricular white matter, bilateral temporal lobes, and right occipital lobe (Fig. 3A, B). CTA demonstrated complete occlusion (100%) of the right posterior cerebral artery (PCA) at the P2 segment (Fig. 4). Chest CT demonstrated bilateral pulmonary inflammation and fibrotic foci. Doppler ultrasound of bilateral lower extremity veins showed no significant abnormalities. Based on the imaging findings, the local hospital suspected CFE following multiple fractures. The patient was subsequently transferred to their intensive care unit (ICU) for further monitoring; however, his symptoms showed no improvement. Due to the critical and complex nature of his condition, he was later transferred to the Neurosurgery ICU at our hospital on the 13th day after the injury.

On admission, vital signs were as follows: temperature 36.8°C, pulse rate 104 beats/min, respiratory rate 16 breaths/min, blood pressure 132/80 mm Hg. The patient presented in a state of mild coma (Glasgow Coma Scale E3V1M3). Both pupils were equal in size and round, approximately 2 mm in diameter, with delayed light reflexes. Eyes were fixed, showing no nystagmus or strabismus. Corneal reflexes were present. Muscle strength could not be assessed in the limbs. The left upper limb exhibited increased muscle tone, while the remaining limbs showed normal muscle tone. Bilateral Babinski reflexes were positive. No petechiae or ecchymoses were observed on the skin.

Laboratory results: platelet count 319 × 10⁹/L, hemoglobin (Hb) 9.7 g/dL, prothrombin time 12.8 seconds, activated partial thromboplastin time 35.2 seconds, fibrinogen 6.19 g/L, plasma D-dimer 25.19 mg/L DDU. Carotid Doppler ultrasound revealed multiple plaque formations in both internal carotid arteries. The electrocardiogram showed no significant abnormalities. Echocardiography demonstrated normal atrial and ventricular dimensions with no segmental wall motion abnormalities or right-to-left shunting. Immediate consultation with a neurology specialist led to a diagnosis of CFE based on clinical history, laboratory findings, and MRI results.

### 2.3. Therapeutic intervention

On the 1st day after admission, the patient suddenly developed dyspnea with a drop in blood oxygen saturation to 60%. Bedside suctioning provided no significant improvement. Due to unexplained hypoxemia, a chest CT was immediately performed, revealing no significant abnormalities. Oxygen therapy was therefore intensified. However, the patient continued to experience recurrent episodes of hypoxemia. A tracheostomy was performed on the 9th day after admission to maintain airway patency and promote pulmonary recovery. Following a multidisciplinary team discussion, the patient was deemed stable and fit for surgery. On the 17th day of hospitalization, under general anesthesia, the patient underwent open reduction and internal fixation for fractures of the right tibia, fibula, and tibial plateau, including plate fixation and allograft bone transplantation. The patient tolerated the procedure well. Postoperative follow-up revealed Hb levels at 7.2 g/dL and D-dimer at 4.31 mg/L DDU. Considering the patient’s multiple underlying conditions, high postoperative bleeding risk, and low Hb, 3.5 units of packed red blood cells were transfused to alleviate hypoxia and prevent hemorrhage. Hb levels subsequently stabilized. Since admission, the patient also received 3 months of comprehensive adjunctive therapy, including continuous insulin aspart infusion, continuous nasogastric administration of butylphthalide soft capsules 3 times daily, and subcutaneous injection of 5000 IU low-molecular weight heparin twice daily.

### 2.4. Outcome

The patient’s clinical condition gradually stabilized. A follow-up brain MRI on the 30th day after admission showed a reduction in the number of infarcted lesions and a mild decrease in DWI signal intensity (Fig. 3C, D). He was transferred out of the ICU on the same day, and hyperbaric oxygen therapy (HBOT) was initiated at a pressure of 2.0 ATA for 90 minutes daily. Three months after admission, the patient exhibited improved consciousness (Glasgow Coma Scale E4, V-tube, M6) and slight recovery of right-sided limb function (muscle strength grade 3). The patient and his family decided to transfer him to a rehabilitation hospital for continued recovery treatment.

## 3. Discussion

Fat particles are detectable in the blood or urine of over 90% of patients with major fractures, but the vast majority do not progress to FES.^[3,4]^ Studies have shown that the incidence of FES in patients with multiple fractures is approximately 1.29%,^[2]^ while CFE is even rarer, constituting only 0.9% to 11% of FES cases.^[5]^ CFE lacks characteristic physical signs and may present with headache, confusion, aggressive behavior, seizures, cortical blindness, dementia, affective apathy, focal deficits (e.g., hemiparesis, aphasia, agnosia), and altered states of consciousness with hallucinations (auditory or sexual), progressing to coma. These symptoms typically emerge 12 to 36 hours post-injury. As demonstrated in this case, the patient developed impaired consciousness the day after sustaining severe multiple fractures. However, when neurological symptoms predominate, their variable and nonspecific nature (particularly in the absence of the classic triad) can easily lead to misdiagnosis as encephalopathy caused by traumatic brain injury, shock, or hypoxia.

The mechanisms by which CFE causes brain injury remain incompletely understood, with prevailing theories grounded in mechanical and biochemical hypotheses.^[3,5,6]^ The underlying mechanisms may include: mechanical obstruction of cerebral microvasculature by fat emboli, leading to focal cerebral ischemia and microinfarction; blood–brain barrier disruption by free fatty acids, resulting in cerebral edema and neurological dysfunction; pulmonary fat embolism causing ventilation/perfusion mismatch and hypoxemia, leading to cerebral hypoxia; systemic inflammatory response triggered by fat emboli, releasing inflammatory mediators that exacerbate cerebral inflammation and injury; activation of the coagulation cascade by fat emboli, promoting platelet aggregation, intravascular coagulation, increased vascular permeability, vasogenic edema, and microhemorrhages; secondary embolism of cerebral small vessels by free fatty acids released upon the dissolution of fat emboli. Notably, in this case, the patient exhibited significant neurological deficits early in the clinical course, unaccompanied by severe respiratory failure. This clinical feature suggests that biochemical injury mediated by free fatty acids (including blood–brain barrier disruption and cerebral inflammation) may have played a more critical role than mechanical obstruction alone in the pathogenesis of brain injury.

FES is primarily diagnosed based on clinical manifestations, with the criteria proposed by^[7]^ Gurd et al in 1974 (Table 1) currently being the most widely applied. No definitive diagnostic criteria exist for CFE, and its diagnosis initially relies mainly on medical history and clinical presentation. However, this case presents with the uncommon initial symptom of isolated impaired consciousness and lacks typical physical signs such as skin ecchymoses, complicating diagnosis.^[5]^ In such diagnostic uncertainties, advances in imaging have established brain MRI as the widely accepted gold standard for diagnosing CFE, particularly DWI, due to its high sensitivity for detecting early lesions.^[8,9]^ MRI features of CFE typically include scattered, multiple lesions in periventricular white matter, subcortical regions, and basal ganglia. DWI sequences reveal a characteristic “starry sky sign” pattern of hyperintensity early in the disease course. In this case, brain MRI + DWI performed immediately after the onset of impaired consciousness revealed typical imaging features of CFE, thereby supporting the preliminary diagnosis. Therefore, for patients with post-fracture impaired consciousness presenting atypical clinical manifestations, even if they do not meet the Gurd diagnostic criteria, high vigilance for CFE should be maintained. Prompt MRI–DWI examination is essential to rule out CFE.

**Table 1:** Gurd and Wilson diagnostic criteria for FES.

Category	Diagnostic criteria
Major criteria	Respiratory insufficiency. Cerebral involvement (unexplained by head injury). Petechial rash.
Minor criteria	Tachycardia pyrexia (fever). Retinal changes (fat or petechiae) jaundice. Renal changes (anuria/oliguria or fat globules in urine). Elevated ESR thrombocytopenia. Fat macroglobulinemia.
Diagnostic requirement	FES is diagnosed by the presence of 1 major criterion and 4 minor criteria.

ESR = erythrocyte sedimentation rate, FES = fat embolism syndrome.

To determine the etiology, we systematically ruled out other common stroke types: first, cranial CT showed no acute hemorrhagic lesions, excluding hemorrhagic stroke; second, despite the patient’s presence of local thrombotic risk factors such as hypertension and diabetes, and despite the right PCA segment P2 showing occlusion on cranial CTA, the infarct distribution was diffuse and bilateral. This presentation is inconsistent with the typical lesion morphology in this vascular territory. No other acute large vessel occlusions were identified, thus ruling out acute large vessel occlusive stroke. Third, carotid ultrasound revealed only atherosclerotic plaques without active thrombus; fourth, the patient had no history of cardiac disease. Admission electrocardiogram and transthoracic echocardiography showed no evidence of potential cardiogenic embolism (e.g., atrial fibrillation, abnormal ventricular wall motion, intracardiac thrombus, or valvular vegetations). Additionally, regarding the patient’s elevated D-dimer levels and multiple infarct foci, we further evaluated other causes of microvascular and perfusion disorders. Fifth, the likelihood of microembolism due to a hypercoagulable state (e.g., disseminated intravascular coagulation) was low, as the patient had normal platelet counts, no significant coagulation abnormalities, and no supporting evidence such as red blood cell fragments. Sixth, hemodynamic cerebral infarction due to dehydration or anemia is also unlikely, as the patient presented without severe hypotension or shock, maintained adequate Hb levels, and the infarct distribution did not align with its typical characteristics (the junctional zone of cerebral artery supply areas). Conversely, extremely elevated D-dimer levels can occur in severe FES, reflecting systemic inflammation and coagulation system activation, supporting the diagnosis of CFE. Based on the above exclusionary diagnoses, the patient’s MRI–DWI showing a classic “starry sky sign,” the non-vascular distribution of lesions, and the clear temporal relationship with multiple fractures strongly support CFE as the primary cause of this cerebral infarction. The patient’s fever, tachycardia, decreased Hb, and markedly elevated D-dimer (reflecting systemic inflammation and coagulation activation associated with FES) further confirm the diagnosis of CFE.

Notably, the imaging presentation in this case exhibited a relatively complex pattern. While the brain MRI–DWI sequences demonstrated the classic “starry sky sign,” they also revealed complete occlusion of the right PCA at the P2 segment. This imaging manifestation of “CFE combined with large vessel occlusion” is clinically rare, with limited reports in the literature. This finding suggests that in complex trauma cases, acute CFE may coexist with chronic, preexisting cerebral vascular lesions. Although a comprehensive analysis indicated the P2 segment occlusion was a chronic lesion inconsistent with the diffuse distribution of the acute infarction, this imaging combination undoubtedly increased diagnostic complexity. Additionally, another noteworthy finding emerged.^[10]^ Despite the absence of a typical “snowstorm sign” on chest CT, the patient exhibited hypoxemia that could not be fully explained by pulmonary infection alone. This suggests a potential association with FES, warranting further exploration.

CFE currently lacks specific therapy, and the core of management lies in early prevention, timely diagnosis, and symptomatic/supportive treatment.^[5,11]^ The patient in this case received a comprehensive treatment regimen including respiratory support, anticoagulation therapy, and HBOT, resulting in favorable clinical and imaging outcomes. In terms of pharmacological management, we carefully weighed the evidence and risks associated with different medications. The decision to use low-molecular weight heparin was based on a dual rationale: first, to address the patient’s severe hypercoagulable state, as evidenced by markedly elevated D-dimer levels (25.19 mg/L) and newly developed deep vein thrombosis, aiming to prevent secondary thromboembolic events; second,^[12]^ heparin may enhance lipase activity, potentially accelerating the clearance of lipids from the bloodstream, thereby contributing to therapeutic efficacy. In contrast,^[13]^ although corticosteroids could theoretically mitigate the inflammatory response associated with FES, their use remains controversial and lacks consistent high-quality evidence supporting routine application. Considering this particular patient’s advanced age, coexisting diabetes, and multiple fractures (factors carrying potential risks of worsened glycemic control and impaired wound healing) we decided not to include corticosteroids in the treatment plan. In terms of adjunctive therapy, HBOT was primarily employed to address the core pathological mechanism of CFE: cerebral hypoxia. For this patient with refractory hypoxemia,^[14,15]^HBOT likely exerted its therapeutic effects through multiple mechanisms, such as improving oxygen delivery to the ischemic penumbra, reducing cerebral edema, and modulating inflammatory responses. Although the efficacy of interventions like heparin and HBOT requires further validation in large-scale trials, the clinical and radiological improvement observed in this complex elderly patient suggests the potential value of this treatment approach.

There are reports in the literature showing that most patients with CFE have a good prognosis and can recover completely within weeks to months.^[16]^ It should be noted, however, that elderly patients, such as the one in this case, often have comorbid hypertension, diabetes mellitus, and underlying cerebrovascular pathology, and neurologic recovery may be slower and incomplete in this population.^[17]^ The underlying diseases not only impair the reserve function and repair capacity of brain tissue but may also increase the risk of complications during treatment. However, the positive outcome of this case suggests that even in the presence of underlying disease, a favorable prognosis may still be achieved through early identification and comprehensive intervention. However, this case also warns clinicians to maintain a more cautious expectation of the recovery process of such patients and to implement individualized long-term rehabilitation strategies.

## 4. Conclusion

This case details an elderly patient with multiple comorbidities who presented with atypical CFE, characterized by isolated impaired consciousness as the initial symptom following multiple fractures. This case highlights the importance of maintaining a high index of suspicion for CFE in patients with multiple fractures who do not present with the typical triad of symptoms. Early brain MRI–DWI examination and identification of the characteristic “starry sky” sign are pivotal for definitive diagnosis. Although this is a single-center case report with limited generalizability, the observed positive outcome suggests that prompt comprehensive supportive care combined with early rehabilitation intervention may contribute to improved prognosis in such complex cases. However, given the ongoing controversy surrounding therapies like heparin and hyperbaric oxygen, future larger-scale studies are warranted to validate these findings and establish evidence-based management guidelines for CFE.

## Acknowledgments

The authors would like to thank the patient for agreeing to the publication of the article.

## Author contributions

**Writing – original draft:** Yu Wei.

**Writing – review & editing:** Hanyu Zhong, Guanghong Xiang.
